# How Reliable Are Hematological Parameters in Predicting Uncomplicated *Plasmodium falciparum* Malaria in an Endemic Region?

**DOI:** 10.1155/2013/673798

**Published:** 2013

**Authors:** Haruna Muwonge, Sharif Kikomeko, Larry Fred Sembajjwe, Abdul Seguya, Christine Namugwanya

**Affiliations:** 1 Department of Medical Physiology, School of Biomedical Sciences, Makerere University College of Health Sciences, P.O. Box 7072, Kampala, Uganda; 2 School of Medicine, College of Health Sciences, Makerere University, P.O. Box 7072, Kampala, Uganda

## Abstract

**Background:**

Malaria remains endemic in Sub-Saharan Africa. Hematological changes that occur have been suggested as potential predictors of malaria. This study was aimed at evaluating the diagnostic relevance of hematological parameters in predicting malaria.

**Methods:**

A cross-sectional study involving 370 patients with signs and symptoms of malaria was conducted at Mulago Hospital, Kampala, from May, 2012 to February, 2013. Thin and thick blood films were prepared for each patient and stained with Giemsa to aid the detection of malaria parasites. Patients’ hematological parameters were determined.

**Results:**

Out of the 370 patients, 61 (16.5%) had malaria. Significant differences in the hematological parameters between *P. falciparum* malaria parasitemic patients and nonparasitemic patients were only observed in mean (±SD) of the differential monocyte count (10.89 ± 6.23% versus 8.98 ± 5.02%, *p* = 0.01) and the platelet count (172.43 (± 80.41) ×10^3^ cells/*μ*l versus 217.82 ± (95.96) ×10^3^ cells/*μ*l *p* = 0.00). The mean (±SD) values of the red blood cell indices (hemoglobin count, MCV, MCH, and MCHC), the differential neutrophil and lymphocyte counts, and the mean platelet volume (MPV) did not significantly differ between the two groups.

**Conclusion:**

Hematological changes are unreliable laboratory indicators of malaria in acute uncomplicated *Plasmodium falciparum* malaria.

## 1. Background

The last decade has witnessed a massive scale up of malaria prevention efforts, primarily through the use of long-lasting insecticide treated bed nets, indoor residual spraying with insecticides, and an increase in accessibility to malaria diagnostic facilities. These measures, among others, are said to have saved an estimate of more than 735,000 lives in 34 African countries over the last 10 years. Yet malaria remains an enormous public health problem, responsible for 781,000 deaths in a year, most of which are African children of less than five years of age [1]. In Uganda, malaria is still the leading cause of illness and deaths, accounting for 25–40% of all outpatient visits at health care facilities, 20% of hospital admissions, and 15% of inpatient deaths [2].

Malaria is caused by a protozoan parasite of the genus: *Plasmodium. P. falciparum*, *P. ovale*, *P. vivax*, and *P. malariae* are the most common species, with *P. falciparum* being the most virulent. Hematological alterations that are thought to characterize malaria may be related to the overt biochemical changes that occur during the asexual stage of the life cycle of the malaria parasite. Entry of *P. falciparum* into erythrocytes usually leads to a marked increase in secretion of inflammatory cytokines (TNF*α*, IL-1, IL-10, and IFN*γ*), endothelial cell activation (due to overexpression of cell adhesion molecules; ICAM-1, VCAM-1), activation of the coagulation cascade (due to platelet consumption and endothelial damage), and sequestration of parasitized RBCs (due to overexpression of cell adhesion molecules, pfEMP, and iNOS [3–7]). These along with other mechanisms set in motion events that ultimately result in morphological and numerical changes in the different blood cell lines.

The World Health Organization recommends that all persons of all ages in all epidemiological settings with suspected malaria should receive a parasitological confirmation of diagnosis [1]. Microscopic detection and identification of plasmodium species in Giemsa stained thick blood films (for screening) and thin blood films (for species confirmation) is the accepted worldwide “gold standard” used for the routine diagnosis of malaria [1, 8]. However, because microscopy requires trained staff, well maintained equipment, a regular supply of reliable reagents, clean water, electricity, and a well-executed quality management system, high quality microscopy services have not been widely available for the diagnosis of malaria in some malaria endemic communities [1]. In many countries, ensuring quality management of microscopy at all levels of the health care system has not been feasible [1].

Therefore, during the past decades, various efforts to replace the traditional blood film for the diagnosis of malaria have revived interests in the possibility of using routine hematological blood parameters to aid the presumptive diagnosis of malaria infection [8]. Alterations in the hematological parameters are also thought to have the capacity to act as an adjuvant tool in strengthening the suspicion of malaria, thereby prompting a more meticulous search for malaria parasites [9]. Although the diagnostic implications of the changes in hematological parameters of patients with severe *P. falciparum* malaria have been clearly mentioned from prior studies, there is still a lack of evidence regarding their diagnostic relevancy in uncomplicated *P. falciparum* malaria. This paper therefore intends to bridge the knowledge gap by providing a report from a study that set out to determine the hematological parameters in patients with acute uncomplicated malaria attending the Mulago hospital outpatients’ clinic, in a malaria endemic area of Kampala (Uganda) so as to ascertain their diagnostic relevance or reliability in predicting malaria.

## 2. Methods

A prospective cross-sectional study was conducted at the Mulago National Referral Hospital in Kampala during and after the rainy season, a period between the month of May 2012 and February 2013.

Mulago hospital is a 1,500-bed hospital serving both as the Uganda national referral as well as the Makerere University college of Health sciences teaching hospital. It is located in the Central division of Kampala, Uganda’s capital. Kampala lies in the south central region of Uganda, which is endemic to malaria. This region has two rainy seasons: February to April and August to November, the rest of the months being relatively dry. In addition to urban and suburban areas, Kampala city has several swampy and unpopular slum areas with poor drainage and waste disposal facilities. These in turn provide fertile breeding grounds for the female anopheles mosquito, the vector for *Plasmodium falciparum*, leading to the burden of endemic malaria.

A total of 370 patients with signs and symptoms highly suggestive of malaria were recruited. For each subject, a written informed consent was obtained. A detailed clinical history was obtained and a thorough examination was done for every subject enrolled for the study. Patients with clear signs or symptoms of a localized infection, for example, an upper respiratory tract infection, urinary tract infection, or other infections, and patients who gave a clear history of receiving antimalarial treatment before presentation to the clinic were excluded.

A sample of 3mLs of venous blood was collected from each participant into ethylene diamine tetra-acetic acid (EDTA) bottles and promptly analyzed for routine hematological parameters which included total white blood cell count (TWBC), differential WBC count percentages for neutrophils, basophils, eosinophils, and monocytes, Hemoglobin level (Hb), red blood cell count (RBC), hematocrit (Hct), mean cell volume (MCV), mean cell hemoglobin (MCH), mean cell hemoglobin concentration (MCHC), red blood cell distribution width (RDW), platelet count (PC), and mean platelet volume (MPV). The hematological parameters were determined using a Coulter A-T Pierce hematology analyzer (Beckman Coulter, Inc. Fullerton, CA, USA). Daily quality assurance checks were performed and recorded, and commercial standards were used in accordance with the manufacturer’s instructions. In this study, anemia was defined as a hemoglobin concentration of <10 g/dL for both male and female subjects, whereas thrombocytopenia was defined as a platelet count of <150,000 cells/*μ*L.

Two slides for microscopy were promptly prepared after specimen collection, each with a thin and thick blood smear. Blood smear slides were stained with Giemsa. Two experienced microscopists examined every slide from a study participant independently, and a consensus was reached in recording the final result. Presence or absence of plasmodia parasites, the plasmodia species, and the number of asexual parasites per 200 WBCs were determined. Parasite density was calculated as the ratio of parasites to WBCs permicroliter of blood using a thick blood film (parasites/WBCs counted × total WBCs in 1 *μ*L of blood).

Data was entered in Microsoft Excel (2010) software program and doubly checked for errors before being exported to IBM SPSS Statistics 19 software for analysis. Patients were categorized based on microscopy results into parasitemic (malaria smear positive) and nonparasitemic (malaria smear negative) groups. Data for the different hematological parameters was expressed as mean (±SD). Multivariate linear regression models were used to assess for any sex differences in the hematological parameters in either parasitemic or nonparasitemic groups. Hematological parameters between different groups were compared using the Student’s *t*-test or Analysis of Variance (ANOVA). A *P* value of <0.05 was considered statistically significant for *t*-test comparisons, where, as a value under Welch’s F (3, 30.17) = 10.95, *P* < 0.001 was statistically significant for comparisons done with ANOVA.

Written informed consent was obtained from all patients. The study was conducted following approval from the Ethical Review Boards of the School of Biomedical Sciences, Makerere University College of Health Sciences, Mulago Hospital, and the Uganda National Council of Science and Technology.

## 3. Results

The study involved 370 suspected cases of malaria infection. The mean age of the participants was 28 years, whereas the male (108) to female (262) ratio was approximately 1 to 2.5 (1 : 2.5). Out of the total number of participants, 16.5% (*n* = 61) had *P. falciparum* malaria confirmed by microscopy, whereas the rest tested negative (*n* = 309). The mean age of the parasitemic and nonparasitemic patients was 26.31 (±9.94) years and 28.25 (±10.14) years, respectively. Therefore, this close age range shows that age was not a confounding factor in our analysis.

On presentation to the clinicians, most of the patients had been ill for about 3–5 days before the first visit. The various symptoms, their frequency, and level of significance (*P* value) are shown in Table 1 for both parasitemic (smear positive) and nonparasitemic (smear negative) groups. As shown in Table 1, there was no significant difference in the clinical presentation between parasitemic and nonparasitemic patients (*P* > 0.05). However, fever plus headache, or generalized body weakness, or joint pains were the commonest presentations in both the parasitemic and nonparasitemic groups.

The mean (±SD) values of the different hematological parameters, stratified for sex, were determined for both malaria parasitemic and nonparasitemic groups. Multivariate linear regression analysis showed no significant sex differences in the different hematological parameters in either the parasitemic or nonparasitemic groups. Hematological parameters for the malaria parasitemic group were then compared with those of the nonparasitemic group using the Student’s *t*-test as shown in Table 2.

The respective mean (±SD) values of the total WBC count, lymphocytes, neutrophils, eosinophils, monocytes, RBC count, hematocrit, hemoglobin, RDW, platelets, and MPV in malaria parasitemic patients versus nonparasitemic patients are shown in Table 2. The mean values for the platelet count and the differential percentage of lymphocytes were both significantly lower for the parasitemic group (172.43 (±80.4) × 10^3^ cells/*μ*L, and 37.38 (±18.54)%) compared with the nonparasitemic group (217.82 (±95.96) × 10^3^ cells/*μ*L and 42.42 ± (15.82)%). Conversely, the differential percentage of monocytes was significantly higher in the parasitemic group (10.89 (±6.23)%) compared to the nonparasitemic group (8.98 (±5.02)%).

Although there were mild differences in the means of the remaining hematological parameters, the differences observed were not statistically significant.

To determine the diagnostic value of the different hematological parameters, the sensitivity, specificity, positive predictive value (PPV), negative predictive value (NPV), and likelihood ratio (LR) were determined after comparison between parasitemic and nonparasitemic groups as shown in Table 3. A hemoglobin level <10 g/dL, RBC < 4 (×10^12^/L), MCV < 80 (fl), RDW > 15 (%), PLT < 150 (×10^9^/L) and MPV < 8 fl had been singled out as the most likely predictors of malaria infection from prior studies [10–12]. As seen in Table 3, low hemoglobin, low red blood cell count, low MCV, low red blood cell distribution width, and low platelet count had fairly good specificities, although the sensitivities were very low.

As shown in Table 4, only 25 (41%) of the 61 patients who tested positive for malaria had a parasite density of >5000 parasites/*μ*L. Higher parasite densities were associated with lower lymphocyte and monocyte counts.

## 4. Discussion

For malaria patients, a prompt and accurate diagnosis is key to effective disease management [13]. Current detection and diagnosis of parasite infections still rely heavily on laboratory methods and/or clinical history, which in most cases is very nonspecific. Before the 1980s, primary tests that had been used to diagnose many parasitic diseases had changed little since the development of the microscope in the 15th century by Antonie Van Leeuwenhoek. However, in the last 3 decades, big strides have been made in refining, modifying, or inventing highly sensitive and specific diagnostic tools for parasitic infections. For malaria diagnosis, these newer tests are based on serology based assays (falcon assay screening test ELISA (FAST-ELISA), and rapid antigen detection systems (RDTs)), molecular based approaches (real time polymerase chain reaction, loop-mediated isothermal amplification (Lamp), and luminex), and proteomics technology (mass spectrometry) [14].

The newer serological and molecular based malaria diagnostic approaches not only provide superior sensitivity and specificity, but also do so at a huge cost, in terms of equipment, infrastructure, and personnel. This makes most of the newer diagnostic methods inapplicable to many areas in Sub-Saharan Africa, where malaria is highly prevalent. Some of the hospitals in the region, however, can afford to carry out a complete blood count for hematological parameters in patients suspected to have an infection. Because microscopy is still considered by many as an imperfect gold standard, efforts have been made to examine the role of hematological parameters in the diagnosis of malaria infection.

The results from this study show that hematological parameters of patients with uncomplicated *P. falciparum* malaria are unreliable indicators for the presence of disease. Previous studies had revealed significant morphological and numerical changes in all the blood cell lines in malaria [9, 11, 15–19]. The changes observed, however, were usually dependent on the parasite species [19–21], disease severity [22] (complicated versus uncomplicated malaria), and the immune status of an individual [12, 23] (person living in a malaria endemic region versus person living in a nonmalaria endemic region), and therefore were found to vary from one person to another or from one region to another.

Malaria is endemic in over 95% areas of Uganda, with the country reported to have the highest incidence rates [24] (478 cases per 1000 people per year) and malaria transmission intensities [25] worldwide. Due to the irregularity in reporting and unavailability of reports, the countrywide prevalence of malaria is unknown. Malaria prevalence data in adults living in Uganda could not be readily accessed at the time this study was done. However, data from anecdotal reports indicates that the prevalence of malaria in under 5-year-old children living in Kampala district is about 5%, with prevalence rates in other regions ranging between 38 and 63% [26]. In this study, out of the 370 patients enrolled with signs and symptoms suggestive of malaria, 61 (16.5%) tested positive for malaria. The percentage of patients who tested positive for malaria was higher in this study than that expected from the region (5%) because the participants enrolled had malaria symptomology and hence a higher likelihood of a positive result following a malaria test.

The clinical presentation of malaria is variable among patients and is usually related to the severity of the infection. The signs and symptoms may overlap with those of other infections. Therefore, assessment of the patient for other possible infections is of paramount importance. Nevertheless, as stated elsewhere [11, 22], majority of patients in this study presented with fever (low or high grade) sometimes with associated chills and rigors, plus generalized body weakness (malaise), joint pains, headache, nausea/vomiting, diarrhea, jaundice, mild dehydration, and conjuctival pallor.

### 4.1. Changes in Hematological Parameters

#### 4.1.1. Changes in Leukocytes

Leukocytes play a vital role in the defense against malaria. Leukocyte changes in malaria are variable and depend on many factors such as acuteness of infection, parasitemia, disease severity, state of the host immunity to malaria, and concurrent infections [22].

Commonly, majority of patients with acute uncomplicated *P. falciparum* malaria usually have their mean total leukocyte count (TLC) within the normal range [18, 22]. However, in some cases, a mild leucopenia may occur, especially in nonimmune adults or in cases of complicated malaria [22, 27]. The mean TLC in parasitemic patients in this study was 5.93 ± 2.72 × 10^3^/*μ*L, which is in agreement with results from prior studies [21]. Nevertheless, despite the fact that the mean TLC in parasitemic patients was normal, an increase in the level of parasitemia in this study was associated with a decrease in the number of leukocytes as shown in Table 2. These findings are similar to those from a retrospective study done in a malaria endemic Indian province involving 334 patients with acute malaria caused by *P. vivax*, *P. falciparum*, or dual infection in which a significant decrease in the mean TLC in the parasitemic group was also observed [11].

In addition, according to previous studies, leukopenia does not appear to be parasite specific as exemplified in a study on patients with *P. vivax* infection in Panama [27], in Turkey (*P. vivax*) [28], and in another study on 404 American service men from Vietnam with *P. vivax*, or *P. vivax*, and *P. falciparum* dual infection [20] where a leukopenia was observed in majority of patients that had malaria caused by several different malaria parasite species.

Overall, the changes seen in the total lymphocyte count in malaria parasitemic patients are usually attributed either to an increase or a decrease in the differential white blood cell (WBC) lines. In consideration of lymphocytes for instance, there have been varying reports from different studies on whether the differential lymphocyte count remains normal, increased, or decreased in an acute malaria infection. Pre-1970s literature had indicated that lymphocyte count remains normal during an acute malaria infection [22]. However, most recent literature shows that lymphopenia, which is sometimes profound but transient or temporary, is a common finding in acute malaria in nonimmune adults [22, 23] as well as in children found in malaria endemic areas [10, 22]. Immunosuppression from HIV-1 infection, for instance, is associated with the development of severe malaria, commonly anemia, cerebral malaria, and a high parasite density [29]. In our study, although the differential lymphocyte count stayed within the normal range for age and sex, it was slightly significantly lower (37.38 (±18.54)%, *P* = 0.03) in *P. falciparum* parasitemic patients as opposed to nonparasitemic controls (42.42 (±15.82)%). The transient malaria lymphopenia, particularly observed in T lymphocytes, is usually attributed to the tissue redistribution of lymphocytes [30] from the free flowing pool to the marginal pool at the endothelial lining of the blood vessels [31], or lymphocyte destruction as a result of Fas-induced apoptosis [32]. Because the rate of lymphocyte destruction in malaria is much higher in children and patients with *P. falciparum* infection (as opposed to *P. vivax*, *P. ovale*, and *P. malariae*), the absolute and the differential WBC counts are low [33]. This could probably explain why the mean total lymphocyte count of parasitemic patients (who were adults and therefore had reduced rates of lymphocyte apoptosis) in this study was normal as opposed to that observed in a similar study that was done in a pediatric population [10].

Part of the body’s innate immune response to blood borne pathogens usually involves activation of effector cells which are either phagocytes (neutrophils and macrophages) or Natural killer (NK) cells. It is not surprising therefore that reticuloendothelial hyperplasia, involving macrophages, is one of the most important early pathological hallmarks in malaria [22]. Hence, monocytosis has been one of the most consistent observations reported from prior studies done on the hematological changes that characterize malaria [10, 15, 19, 34]. These findings are in agreement with our study, where a significant (*P* = 0.01) mild monocytosis was observed in parasitemic patients (10.89 (±6.23)%) compared to the nonparasitemic patients (8.98 (±5.02)%).

The mean neutrophil count was normal for both parasitemic (46.28 (±18.30)%) and nonparasitemic patients (42.87 (±15.77)%) in this study. These findings are similar to those from two studies: one involving 400 cases in a malaria endemic region of India, in which about 85% of the patients had normal neutrophil counts [19] and another in Singapore where majority of the adults with acute uncomplicated malaria had normal neutrophil counts [35]. In contrast, though, some earlier studies had reported neutropenia [31] or neutrophilia [15] among malaria cases, especially in the pediatric patients [10]. The mechanism of neutropenia in malaria has been postulated to involve increased margination and sequestration of neutrophils [31] as a result of the increased expression of cell adhesion molecules (ICAM-1 and VCAM-1) that occurs in malaria [6].

The eosinophil count was not significantly different between the parasitemic and nonparasitemic patients in this study (*P* = 0.72). A few other studies that looked at eosinophils in malaria found low levels (eosinopenia) in majority of patients [16], although the significance of these findings was unknown. However, followup of these patients’ days or weeks after treatment surprisingly revealed a marked elevation in the eosinophil count [16], a feature that the researchers attributed to the rebound eosinophilic response that resulted from of an enhanced T helper-2 response that occurred during the malaria recovery period.

#### 4.1.2. Changes in Platelets

Platelets and coagulation factors are vital components of the extraordinary complex environment that surrounds flowing or sequestered parasitized RBCs and the enclosing tubular vascular endothelium [22]. Because of that, a lot of research work has been dedicated to determining the effects of malaria on platelet homeostasis. What is now apparent from those studies is the fact that thrombocytopenia is a major complication of malaria [10, 11, 19, 21, 36], the magnitude of which is dependent on the parasite species or disease severity. In light of the above, *P. vivax* malaria infection and severe malaria have been associated with a more heightened and severe thrombocytopenia than *P. falciparum* infection and uncomplicated malaria. In this study, although the mean platelet count in parasitemic patients (172.43 (±80.41) × 10^3^/μL) was normal, it was significantly (*P* = 0.00) lower than that of the nonparasitemic group (217.82 (±95.96) × 10^3^/μL). This only reiterates the fact that acute uncomplicated malaria is not associated with a marked reduction in platelets, as compared to severe malaria.

In an attempt to compensate for the low absolute platelet count, the bone marrow increases the formation of megakaryocytes, which usually escape from the bone marrow as mega platelets during an acute malaria infection. Evidence to support this hypothesis comes from a study by Kreil et al. [37] that found a marked elevation in the level of thrombopoietin, a key platelet growth factor in patients with malaria. Because of an increase in the amount of mega platelets, the mean platelet volume is increased during an acute malaria infection [10, 11]. In contrast, the mean platelet volume (MPV) of parasitemic patients in this study was normal. These findings may suggest that uncomplicated malaria is associated with mild or nonsignificant changes in the platelet profile.

The pathogenesis of thrombocytopenia is thought to involve a constellation of processes, some of which include splenic pooling of platelets, antibody (IgG) mediated platelet destruction, adenosine diphosphate (ADP) release following the hemolysis of parasitized RBCs, dysmegakaryopoiesis, platelet aggregation and activation, parasite invasion of platelets, platelet phagocytosis, platelet adhesion to erythrocytes, and oxidative stress [22, 38]. The relatively diverse causative pathophysiological mechanisms could probably explain why changes in platelet homeostasis are more prominent than in other blood cell lines. Nevertheless, thrombocytopenia in malaria is observed to improve with disease resolution, and a normal platelet count is usually reported within 7 days after the initiation of antimalarial treatment [21, 39].

#### 4.1.3. Changes in Red Blood Cells (RBCs) and RBC Indices

For continued survival and reproduction, plasmodium parasites need to infect the red blood cells of their human host. Consequently, changes in the red blood cell indices are some of the commonest observations seen in malaria. Anemia, which is a fall in hemoglobin level below the normal range for age, sex, race, or pregnancy status, is the most frequent outward manifestation of such changes. Malaria is the most common cause of severe anemia in endemic areas [22].

Anemia in malaria is believed to occur due to hemolysis of parasitized and nonparasitized RBCs, peripheral removal/sequestration of RBCs, and ineffective erythropoiesis (due to high circulating tissue necrotic factor (TNF*α*)) [19, 22]. In malaria endemic areas, the prevalence and severity of anemia are usually determined by a number of interacting factors. These include, among others, the parasite species, level of parasitemia, age of host, host genetic factors (e.g., coexisting RBC polymorphisms like hemoglobinopathies, G6PD), and nonmalarial causes of anemia (e.g., infections, malnutrition) [22].

As observed elsewhere [11], the mean red blood cell indices (Hb, MCV, MCH, MCHC, and RDW) of patients with acute uncomplicated malaria in this study were normal. This could probably have been because uncomplicated malaria is associated with milder biochemical changes, for example, a lower production of cytokines, less endothelial cell activation, milder changes in the coagulation profile, less sequestration, and less hemolysis as opposed to complicated/severe malaria.

#### 4.1.4. Diagnostic Accuracy of Hematological Parameters in Acute Uncomplicated Malaria

Previous studies involving patients with complicated malaria had demonstrated that a reduced platelet count, reduced white blood cell counts, and decreased red blood cell indices had relatively good sensitivities and specificities in predicting the presence of malaria infection [10, 11]. However, results from our study showed low sensitivities to all the hematological parameters. This could be accounted for by the relatively low sample size that had deranged parameters in the malaria parasitemic group in this study, a fact that was probably related to the reduced disease severity that normally characterizes uncomplicated malaria. Another factor that could have played a part is the fact that patients in this study had uncomplicated malaria infection, as opposed to other studies in which high sensitivities and specificities were reported amongst patients with severe *P. falciparum* malaria infection [10, 11]. Nevertheless, hematological parameters in the parasitemic group in this study did not markedly differ from those of the nonparasitemic group. This therefore reinforces the lack of significance hematological parameters could have in predicting acute uncomplicated malaria infection.

In conclusion, *P. falciparum* uncomplicated malaria does not produce significant changes in the total WBC count, differential WBC (lymphocytes, neutrophils, and eosinophils) count, and RBC indices (Hb, MCV, MCH, and MCHC). However, mild changes were observed in the levels of platelets and monocytes, with the platelet count being inversely proportional to the level of parasitemia, although the differences were not diagnostically relevant because of very low sensitivities. These observations could possibly be explained by a milder biochemical reaction (lower cytokines, mild activation of endothelial cells, and coagulation cascade) that is thought to characterize uncomplicated malaria. The major limitation of this study was the smaller number of patients with deranged hematological parameters especially in the parasitemic group, which created a slightly higher margin of error in the determination of the diagnostic relevance of the deranged parameters. Nevertheless, generally, hematological parameters were found to be unreliable predictors of disease in patients with uncomplicated malaria in a malaria endemic area like Kampala.

## Figures and Tables

**Table 1:** Comparison of signs and symptoms between parasitemic (smear positive) and nonparasitemic (smear negative) patients.

Parameters	Smear negative (*n* = 309) Number (%)	Smear positive (*n* = 61) Number (%)	*P* value
Vomiting	87 (28.2)	24 (39.3)	0.058
Headache	280 (90.6)	59 (96.7)	0.085
Joint pains	252 (82.6)	53 (86.9)	0.21
Chills and rigors	218 (70.6)	40 (65.6)	0.27
Diarrhea	74 (23.9)	14 (23.0)	0.51
Body weakness	286 (92.6)	57 (16.6)	0.53
Sclera of eyes (jaundice)	16 (5.2)	4 (20.0)	0.43
Pallor of conjunctiva	20 (6.5)	4 (16.7)	0.58
Dry tongue	114 (36.9)	22 (36.1)	0.51

At 95% confidence interval, a *P* value < 0.05 was considered significant.

**Table 2:** Comparison of hematological parameters between parasitemic (smear positive) and non-parasitemic (smear negative) patients.

Parameters	Smear negative (*n* = 309) Mean ± SD	Smear positive (*n* = 61) Mean ± SD	Mean difference (SE)	95% Confidence interval (CI)	*P* value
Age (years)	28.25 ± 10.14	26.31 ± 9.94	1.94 (1.42)	−0.84–4.72	0.17
RBC × (10^6^/*μ*L)	4.76 ± 0.67	4.70 ± 0.58	0.06 (0.09)	−0.12–0.24	0.53
Hb (g/dL)	13.61 ± 1.97	13.36 ± 1.63	0.25 (0.27)	−0.28–0.78	0.35
HCT (%)	40.21 ± 5.73	40.08 ± 5.14	0.12 (0.79)	−1.43–1.67	0.88
MCV (fl)	84.84 ± 9.13	85.71 ± 9.21	−0.08 (1.28)	−3.39–1.64	0.50
MCH (pg)	28.72 ± 3.04	28.55 ± 2.84	0.17 (0.42)	−0.66–1.00	0.68
MCHC (g/dL)	33.89 ± 1.96	33.41 ± 2.00	0.47 (0.28)	−0.07–1.01	0.09
RDW (%)	14.17 ± 3.09	14.35 ± 3.00	−0.18 (0.43)	−1.03–0.66	0.67
PLT × (10^3^/*μ*L)	217.82 ± 95.96	172.43 ± 80.41	45.4 (13.11)	19.59–71.18	0.00*
MPV(fl)	10.28 ± 1.52	10.68 ± 1.37	−0.39 (0.22)	−0.83–0.04	0.07
Total WBC × (10^3^/*μ*L)	6.41 ± 4.28	5.93 ± 2.72	0.48 (0.57)	−0.64–1.60	0.40
Neutrophils (%)	42.87 ± 15.77	46.28 ± 18.30	−3.41 (2.28)	−7.89–1.07	0.14
Lymphocytes (%)	42.42 ± 15.82	37.38 ± 18.54	5.04 (2.28)	0.54–9.52	0.03*
Monocytes (%)	8.98 ± 5.02	10.89 ± 6.23	−1.91 (0.73)	−3.35–0.47	0.01*
Eosinophils (%)	3.65 ± 4.15	3.88 ± 5.95	−0.23 (0.63)	−1.47–1.01	0.72
Basophils (%)	1.95 ± 2.24	1.56 ± 1.56	0.39 (0.30)	−0.19–0.98	0.19

* *P* value < 0.05 was significant at 95% confidence interval.

RBC: red blood cells; Hb: hemoglobin; HCT: hematocrit; MCV: mean corpuscular volume; MCH: mean cell hemoglobin; MCHC: mean cell hemoglobin concentration; RDW: red blood cell distribution width; PLT: platelets; MPV: mean platelet volume; WBC: white blood cells.

**Table 3:** Sensitivity, specificity, predictive value, and odds of the different hematological parameters.

Hematological parameter	Nonparasitemic (*n* = 309)	Parasitemic (*n* = 61)	Sensitivity	Specificity	PPV	NPV	LR	*P* value
Hb < 10(g/dL)	12	1	16.0	96.1	7	83.1	0.342	0.703
RBC < 4(×10^12^/*μ*L)	39	7	11.5	87.4	17.9	83.3	0.802	1
MCV < 80	77	15	24.6	75.1	16.3	83.4	0.975	177
RDW> 15 (%)	83	17	27.9	73.1	17	83.7	0.872	0.875
PLT < 150 (×10^3^/*μ*L)	75	26	42.6	75.7	25.7	86.9	0.005	0.005*

* *P* value < 0.05 was significant at 95% confidence interval.

PPV: positive predictive Value; NPV: negative predictive value; LR: likelihood ratio; CI: confidence interval; RBC: red blood cell; MCV: mean corpuscular volume; RDW: red blood cell distribution width; PLT: platelet count.

**Table 4:** Mean ± SD of haematological parameters at different levels of parasitemia.

Parameters	<1000 Parasites (*n* = 9)	1001–5000 Parasites (*n* = 18)	>5000 Parasites (*n* = 34)	*P* value
RBC × (10^6^/*μ*L)	4.66 ± 0.61	4.88 ± 0.59	4.58 ± 0.54	0.28
Hb (g/Dl)	13.77 ± 1.60	13.59 ± 1.48	13.05 ± 1.76	0.413
HCT (%)	40.62 ± 4.90	41.06 ± 5.47	39.42 ± 5.08	0.176
MCV (fl)	87.62 ± 10.60	84.39 ± 7.55	85.90 ± 9.77	0.687
MCH (pg)	29.71 ± 3.05	28.02 ± 2.60	28.52 ± 2.89	0.352
MCHC (g/dL)	34.00 ± 2.14	33.32 ± 2.34	33.31 ± 1.80	0.000*
RDW (%)	12.87 ± 2.26	13.94 ± 2.91	14.95 ± 3.12	0.196
PLT × (10^3^ *μ*L)	179.89 ± 62.72	178.61 ± 82.99	167.18 ± 84.79	0.853
MPV (fl)	9.71 ± 1.40	11.22 ± 1.08	10.65 ± 1.39	0.023
WBC × (10^3^ *μ*L)	6.43 ± 2.43	5.89 ± 3.76	5.70 ± 2.15	0.837
Neutrophils (%)	43.47 ± 19.56	37.15 ± 20.25	51.85 ± 15.02	0.017
Lymphocytes (%)	43.87 ± 19.58	46.97 ± 21.50	30.58 ± 13.60	0.016
Monocytes (%)	6.22 ± 3.87	8.49 ± 4.28	13.41 ± 6.47	0.001*
Eosinophils (%)	4.02 ± 5.10	5.65 ± 7.32	2.91 ± 5.27	0.290
Basophils (%)	2.40 ± 2.60	1.73 ± 1.54	1.24 ± 1.12	0.122

Welch’s F (2, 31.009) = 12.634,

* *P*< 0.001 considered significant.

RBC: red blood cells; HbL: hemoglobin; HCT: hematocrit; MCV: mean corpuscular volume; MCH: mean cell hemoglobin; MCHC: mean cell hemoglobin concentration; RDW: red blood cell distribution width; PLT: platelets; MPV: mean platelet volume; WBC: white blood cells.
